# Tell me something I don’t know

**DOI:** 10.7554/eLife.15853

**Published:** 2016-04-19

**Authors:** Jonas Obleser

**Affiliations:** Department of Psychology, University of Lübeck, Lübeck, Germanyjonas.obleser@uni-luebeck.de

**Keywords:** perception, predictions, surprise, prediction error, predictive coding, auditory cortex, Human

## Abstract

The roles that neural oscillations play in the auditory cortex of the human brain are becoming clearer.

**Related research article** Sedley W, Gander PE, Kumar S, Kovach CK, Oya H, Kawasaki H, Howard MA, Griffiths TD. 2016. Neural signatures of perceptual inference. *eLife* **5**:e11476. doi: 10.7554/eLife.11476

**Image** Detailed analyses of electrical signals in the brain are telling us more about how we perceive sounds

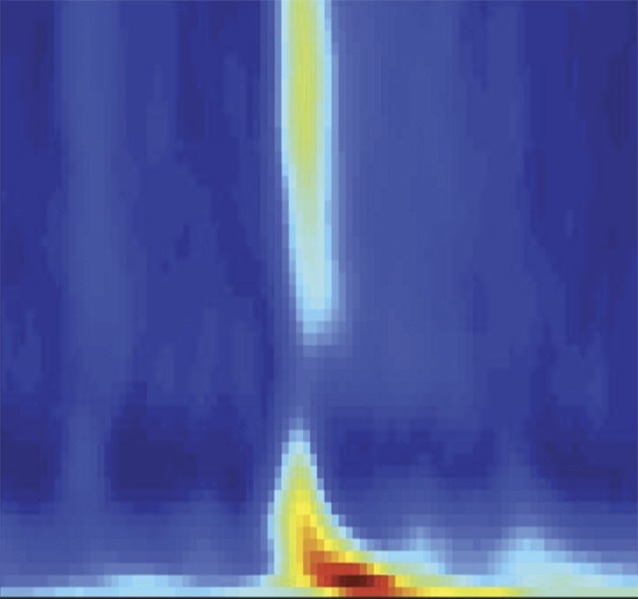

Do you like surprises? If you don’t, it might be because our nervous system works very hard to avoid being surprised. This often involves the nervous system trying to predict or “model” its own future as accurately as possible. For example, when we are listening to a string of sounds that appears to be unpredictable, such as a Charlie Parker-esque saxophone solo, our brain will still try to predict what the next note will be (Figure 1A).

**Figure 1.**
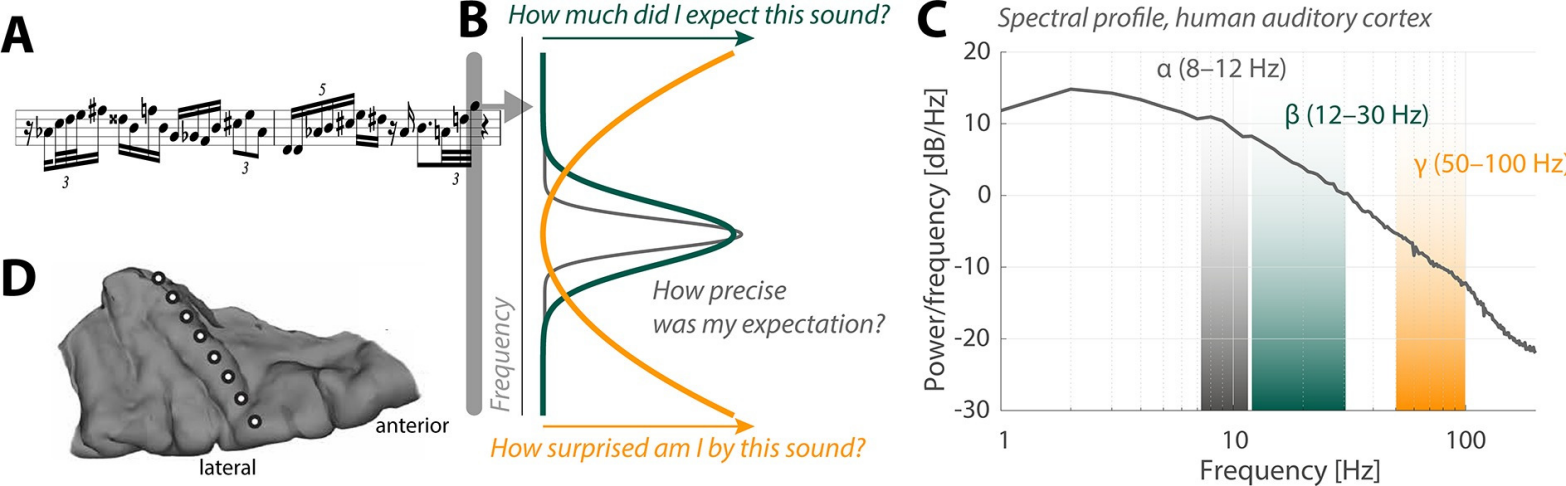
Neural oscillations and the perception of sounds. (**A**) Sensory input in the form of a bebop-style saxophone improvisation (courtesy of Jakob Obleser). (**B**) Schematic representation of the three predictive-coding parameters modeled by Sedley et al.: prediction (green), the precision of a prediction (grey), and surprise (orange). (**C**) Graph showing the strength of neural oscillations (y-axis) as a function of frequency (x-axis) in a region of the auditory cortex called Heschl’s Gyrus. Beta oscillations (green band) code for predictions; alpha oscillations (grey) code for the precision of these predictions; and gamma oscillations (orange) code for surprise. (**D**) The neural oscillations were recorded by placing electrocorticographical depth electrode contacts at different positions (white circles) along Heschl’s Gyrus.

So-called generative models of sensory perception have put forward the idea that the nervous system relies on predictions instead of detailed information about each new sound or other sensory event (Friston, 2010). Under such a regime, the task at hand for a sensory neuron is to filter the sensory information it receives so that it only passes on the information that is truly new – the surprising bits – to other "target" neurons. In modeling terms, this goes by the name of predictive coding . Surprise, in this context, compares the prediction error (that is, the difference between the actual sound and the predicted sound) with the precision of the prediction (that is, how sure were we about our prediction; Figure 1B). Since we need a prediction in order to be able to quantify surprise, this means that the target neurons must somehow tell the sensory neurons what information they are expecting to receive in the first place.

Patterns of rhythmic activity in neurons known as neural oscillations are important to perception. These are exciting times for researchers working on neural oscillations because a framework that describes their specific contributions to perception is finally emerging. In short, the idea is that comparatively slow neural oscillations, known as “alpha” and “beta” oscillations, encode the predictions made by the nervous system. Therefore, alpha and beta oscillations do not communicate sensory information per se; rather, they modulate the sensory information that is relayed to the brain. Faster “gamma” oscillations, on the other hand, are thought to convey the degree of surprise triggered by a given sound.

Now, in eLife, William Sedley and colleagues at Newcastle University and the University of Iowa report the results of work that sheds new light on the roles played by alpha, beta and gamma oscillations in the auditory cortex of the human brain (Sedley et al., 2016). Three patients undergoing neurosurgical treatment for epilepsy provided the researchers with a rare opportunity to directly measure neural oscillations in the auditory cortex with a technique called electrocorticography. The patients listened to a meandering stream of complex tones that would sound random to most people: however, it sounds far from random to the pattern recognition device that is our brain.

Sedley et al. surveyed the neural oscillations in the patients to extract three parameters associated with models of perception: prediction, precision and surprise (Figure 1C,D). Their approach fused the power of statistical models with the unique signal quality of electrocorticography. Each event in the stream of sounds played to the patients was pulled from a large variety of possible sounds, which allowed the researchers to fine-tune the degree of surprise, essentially yielding a new subdiscipline of model-based electrocorticography. Sedley et al. found that gamma oscillations do indeed encode the degree of surprise triggered by a given sound.

To date frameworks for predictive coding have been largely based on studies of the visual domain in non-human primates (see Bastos et al., 2012; 2015; Buschman and Miller, 2007; van Kerkoerle et al., 2014). Research on the auditory cortex has mostly focused on the slow “delta” and “theta” oscillations, because these match the time scales found in speech and music (see Giraud and Poeppel, 2012; Obleser et al., 2012; Lakatos et al., 2013). The results of Sedley et al. finally provide us with evidence that supports the existence of similar predictive coding frameworks in human auditory cortex. Reassuringly, there does not appear to be a fundamental difference in the role played by alpha and beta oscillations in the auditory cortex and the role they play in the better-studied visual domain.

Interestingly, alpha oscillations have recently been tied to rhythmic changes in the degree to which a neuron boosts or reduces information about a sound (Kayser et al., 2015). Fittingly, the work of Sedley et al. now tells us that alpha oscillations encode the precision of predictions in auditory cortex (Figure 1B). This is a very telling observation that moves us beyond what the current predictive coding models would have made us expect.

So what should we take from the work of Sedley et al.? The observed effects of prediction and surprise on neural activity are moderate at most; for example, surprise explained only very small portions of the changes in gamma oscillations in each patient. However, Sedley et al. restricted themselves to attractive, simple-to-interpret linear measures (for example, “more surprise leads to an increase in gamma power”). Thus, these data probably provide a conservative estimate of how accurate populations of neurons in the auditory cortex encode prediction and surprise.

The question still remains as to which aspects of neural communication we are missing by not being able to routinely record neural activity directly from the surface of the human brain. So we are surely in for more surprises, whether we like it or not.
